# Reduction of Adherence of *E. coli* O157:H7 to HEp-2 Cells and to Bovine Large Intestinal Mucosal Explants by Colicinogenic *E. coli*

**DOI:** 10.5402/2011/697020

**Published:** 2011-12-11

**Authors:** A. I. Etcheverría, G. H. Arroyo, R. Alzola, A. E. Parma

**Affiliations:** ^1^Laboratorio de Inmunoquímica y Biotecnología, Departamento de Sanidad Animal y Medicina Preventiva, Facultad de Ciencias Veterinarias, Universidad Nacional del Centro Provincia de Buenos Aires, Pinto 399 (7000), Tandil, Argentina; ^2^Laboratorio de Histología, Departamento de Ciencias Biológicas, Facultad de Ciencias Veterinarias, Universidad Nacional del Centro Provincia de Buenos Aires, Pinto 399 (7000), Tandil, Argentina

## Abstract

Enterohemorrhagic *E. coli* strains (EHEC) had emerged as foodborne pathogens and cause in human diarrhea and hemolytic-uremic syndrome. Because of the widespread distribution of EHEC serotypes and O157 and non-O157 in cattle population, its control will require interventions at the farm level such as the administration of probiotics that produce inhibitory metabolites. *E. coli* O157:H7 shows tissue tropisms for the gastrointestinal tract (GIT) of cattle. The aim of this study was to test the ability of a colicinogenic *E. coli* (isolated from bovine) to reduce the adherence of *E. coli* O157:H7 to HEp-2 cells and to GIT of cattle. We inoculated HEp-2 cells and bovine colon explants with both kinds of strains. Colicinogenic *E. coli* was able to reduce the adherence of *E. coli* O157:H7 to HEp-2 cells and to bovine tissues.

## 1. Introduction

Enterohemorrhagic *E. coli* strains (EHEC) are a subset of Shiga toxin-producing *E. coli* (STEC) that had emerged as foodborne pathogens [1–3]. Human illnesses caused by EHEC range from watery diarrhea or hemorrhagic colitis to the hemolytic-uremic syndrome or thrombotic thrombocytopenic purpura [4]. The predominant EHEC serotype associated with the most severe disease is O157:H7 [2, 5]. Various studies confirmed that cattle are the main reservoir of STEC [6–15].

The persistence of *E. coli* O157:H7 in cattle may be due to the ability of the bacteria to colonize a particular location within the gastrointestinal tract (GIT). Several authors have reported that *E. coli* O157:H7 shows tissue tropisms for the colon, lymphoid follicle-dense mucosa at the terminal rectum, and the rectoanal junction [16–18]. *E. coli* O157:H7 intimately attaches to a variety of cell types and tissues, and a few studies have demonstrated that it can form attaching and effacing lesions on explants of bovine intestinal tissues [19, 20].

Because of the widespread distribution of EHEC serotypes, O157, and non-O157, in cattle population, its control will require interventions at the farm level [21]. A promising method for the control of foodborne pathogens in livestock is the feeding of beneficial bacteria, often referred to as probiotics [22]. Probiotics can interfere with pathogenic strains by producing metabolites that are inhibitory to *E. coli* O157:H7. Some strains of *E. coli* can produce colicins that are inhibitory *in vitro* to diarrheagenic *E. coli* strains, including O157:H7 [23]. Several authors have identified bacteria with potential ability to exclude *E. coli* O157:H7 from the GIT of cattle [23–25]. In a previous study, we isolated strains of colicinogenic* E. coli* from bovine colon which have the ability to inhibit the growth of *E. coli* O157:H7 *in vitro* [26]. Taking into account this fact, the aim of this study was to test the ability of colicinogenic* E. coli* to interfere with the adherence of *E. coli* O157:H7 to HEp-2 cells and to bovine colonic explants.

## 2. Materials and Methods

### 2.1. Bacterial Strains

A strain of *Escherichia coli* O157:H7 (*stx_2_*, *eae, ehxA*) isolated from grazing cattle and a colicinogenic *E. coli* with anti-O157 activity isolated previously by us [26] were used to prepare the inocula. Colicinogenic *E. coli* used in this study was selected taking into account the size of the inhibition zone and the inhibitory activity against different *E. coli* serotypes (O20:H19; O25:H19; O91:H21; O113:H21; O117:H7; O145:H-; O171:H2; O174:H21; O175:H8) isolated in our laboratory in previous work. *E. coli* O157:H7 was selected based onto its virulence genotype, which is the frequently found in HUS-producing O157:H7 isolates. Cultures of both strains were grown overnight on Luria Bertani broth (LB), with shaking (200 rpm) at 37°C. The cultures were washed twice with phosphate-buffered saline and adjusted at a concentration of 2 × 10^7^ cfu mL^−1^. Both strains were resistant to nalidixic acid (50 *μ*g mL^−1^).

### 2.2. Culture of HEp-2 Cells

The cell line was kindly provided by INTA Castelar. Cells were cultured in Minimal Essential Medium (MEM 0643 Sigma) added with 10% of fetal calf serum (Internegocios S.A.) at 37°C with 5% CO_2_.

### 2.3. Inoculation of HEp-2 Cells

For the adherence assays, we used 24 well tissue culture plates (Corning 25820).

The supernatant was discarded and plates were washed three times with phosphate-buffered saline (PBS), then it was added fresh medium in each well.

For inoculating the cells, *E. coli* O157:H7 was cultured in Luria Bertani broth at 37°C for 18 h with shaking, the culture was adjusted by OD_600_ to a concentration of 10^5^ cfu mL^−1^, and the culture of colicinogenic *E. coli* was adjusted to two different concentrations (10^5^ and 10^6^ cfu mL^−1^). We inoculated 100 *μ*L of the suspension of *E. coli* O157:H7 alone, 100 *μ*l of each colicinogenic *E. coli* suspension alone, and two different mixtures: (i) *E. coli* O157:H7 (10^5^ cfu mL^−1^, 100 *μ*L) plus colicinogenic *E. coli* (10^5^ cfu mL^−1^, 100 *μ*L) and (ii) *E. coli* O157:H7 (10^5^ cfu mL^−1^, 100 *μ*L) plus colicinogenic *E. coli* (10^6^ cfu mL^−1^, 100 *μ*L).

Plates were incubated at 37°C in 5% CO_2_ during 3 h, the monolayer was washed three times with PBS, and 100 *μ*L per well of Trypsine-EDTA was added. The supernatants with the cell disattached were recovered to be seeded onto agar plates. Several dilutions of the cell suspensions were seeded onto MacConkey Sorbitol agar plates supplemented with 50 *μ*g *μ*L^−1^ nalidixic acid (SMAC-NAL plates) to quantify sorbitol-negative and sorbitol-positive colonies corresponding to *E. coli* O157 and colicinogenic *E. coli,* respectively.

The experiments were performed in triplicate.

### 2.4. Collection of Explants

Sections of 10 cm of bovine colon were obtained at slaughter immediately after killing. Tissues were washed with Minimal Essential Medium (MEM 0643) and transported to the laboratory on ice. Prior to the inoculation, fat was removed and tissues were opened along the mesenteric border and placed in cold MEM. The tissues were washed 3 times for a period of 10 min each. Then, they were washed with 0.9% NaCl during 30 min with shaking. The samples were placed in MEM without antibiotics. The tissues, now referred as explants, were cut into 3 × 5 mm pieces which were placed mucosal side up onto sterile sponges with two explants per sponge. They were placed in each well of 6 well tissue culture plates (Greiner Bio-One 657 160).

### 2.5. Inoculation of Explants

Each explant was inoculated with 25 *μ*L of bacterial suspensions containing 2 × 10^7^ cfu. One explant was inoculated with *E. coli* O157:H7 only, another one with colicinogenic *E. coli,* and the last one with *E. coli* O157:H7 and colicinogenic *E. coli* equally. We left an explant without inoculating as negative control. The explants were incubated in MEM for 6 h at 37°C in 5% CO_2_ atmosphere on a rocker. MEM was added until it just reached the base of the explants. During the incubation, the medium was replaced hourly by fresh sterile one to avoid the overgrowth of bacteria and maintain constant pH.

### 2.6. Processing of Explants

After the incubation, each explant was cut in half. One piece of each was processed for culture in SMAC-Nal plates, and the other was fixed in 10% neutral buffered formalin and processed for paraffin sectioning according to standard techniques. Sections from each paraffin block were stained immunohistochemically for the detection of O157:H7 adherent bacteria. These sections were firstly incubated with rabbit anti-*E. coli* O157 serum as the primary antibody in a 1 : 200 dilution with Triton X-100-carrageenan (TCT). Then, a second incubation was given with goat anti-rabbit antibody for 30 min followed by incubation with peroxidase-antiperoxidase complex. To make the reaction visible the sections were incubated with Tris-HCl containing diaminobenzidine and hydrogen peroxide. The sections were observed by light microscopy.

For culture in agar plates, explants were vigorously vortexed in 1 mL of sterile 0.9% NaCl to dislodged attached bacteria, and 100 *μ*L of these suspensions were plated onto SMAC-Nal plates and incubated for 18 h at 37°C. Sorbitol-negative colonies were tested with a latex agglutination test for *E. coli* O157 (*E. coli* O157 Latex Test Kit-Oxoid). The experiments were performed in duplicate.

## 3. Results

### 3.1. Adherence to HEp-2 Cells

When the adherence of *E. coli* O157:H7 and colicinogenic *E. coli* was evaluated, it was found that depathogenic strain adhered less to HEp-2 cells than the colicinogenic *E. coli* (38.5% versus 68.1% resp.) when both strains were used in equal concentrations. These differences were even higher (3.6% for *E. coli* O157:H7 versus 96.4% for colicinogenic *E. coli*) when the concentration of the colicinogenic strain was ten-fold increased (Table 1). The percentage of adhered bacteria is the result of the average of three independent experiments. The adherence of each strain separately was performed previously, and they showed the same rate of adherent bacteria to HEp-2 cells.

### 3.2. Adherence to Explants

On SMAC-Nal plates seeded from explants inoculated only with either *E. coli* O157:H7 or the colicinogenic strain, we observed both sorbitol-negative and sorbitol-positive colonies corresponding to the pathogenic and colicinogenic *E. coli,* respectively. On SMAC-Nal plates, seeded from explants inoculated with colicinogenic *E. coli* and O157:H7, we obtained only sorbitol-positive colonies corresponding to the colicinogenic strain. The explants without inoculation did not show any colony on the agar plates.

**Figure 1:**
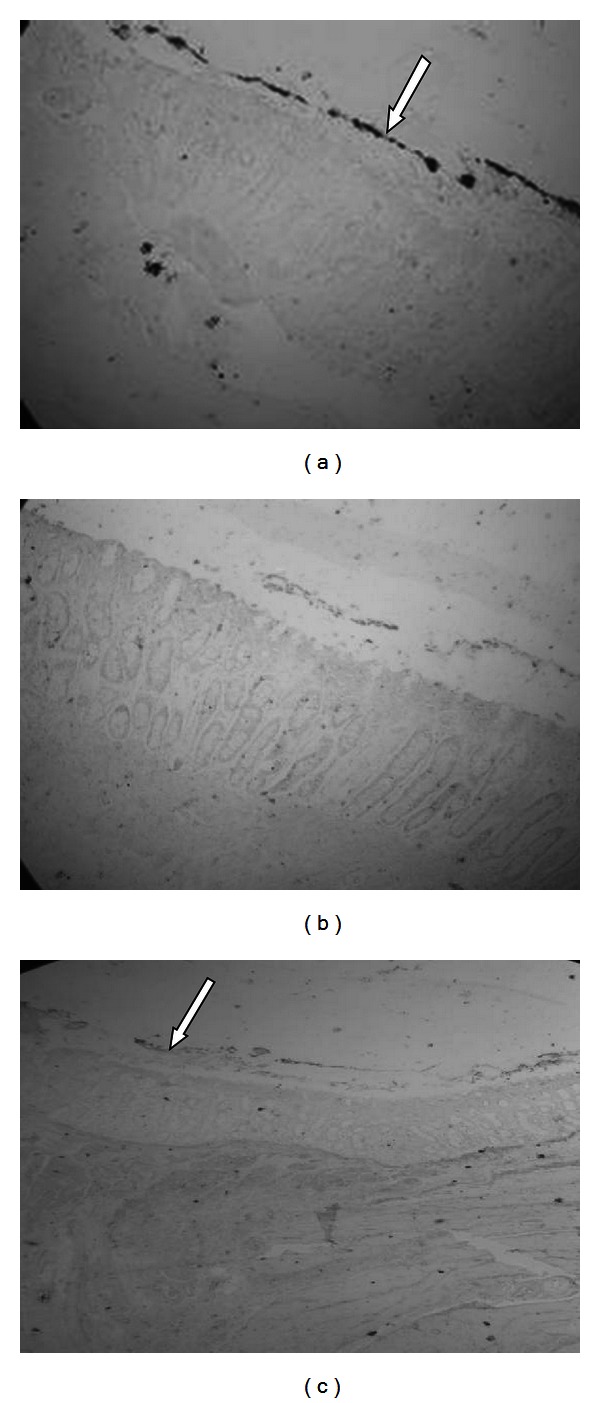
Sections of explants from bovine colon (arrows indicate *E. coli* O157:H7 attached). (a) Bovine colon explant inoculated with *E. coli* O157:H7. Bacteria attached to epithelial edge (10X), (b) bovine colon explant inoculated with colicinogenic* E. coli* (10X), and (c) bovine colon explant inoculated with *E. coli* O157:H7 and colicinogenic* E. coli* (4X).

When the explants inoculated with *E. coli* O157:H7 were analyzed immunohistochemically, we observed staining all along the epithelial edge which corresponds with the pathogenic bacteria attached to colonic epithelium (Figure 1(a)). In the explants inoculated only with the colicinogenic strain, it was not observed any staining (Figure 1(b)). On explants inoculated with a mixture of pathogenic and colicinogenic *E. coli,* we observed a significantly reduction of *E. coli* O157 adhered when compared with explant inoculated only with pathogenic bacteria (Figure 1(c)).

## 4. Discussion

An alternative to reduce the contamination of foods with STEC is the inhibition of proliferation of these pathogenic strains within the ruminant gastrointestinal tract which may be mediated by the use of probiotic bacteria [27]. These beneficial bacteria will fill the same ecological niche of the foodborne pathogens in the gastrointestinal tract, produce inhibitory substances, or modify the microenvironment of the intestinal tract in such a way that is inhibitory or deleterious to the target pathogens [28]. The use of colicinogenic *E. coli* to reduce *E. coli* O157:H7 in cattle is a promising method to control this foodborne pathogen. *E. coli* O157:H7 can itself be colicinogenic, which would make it resistant to certain colicins. However, the use of multiple colicinogenic strains might may reduce this possibility [29]. For that reason, we studied the ability of a colicinogenic *E. coli* strain with anti-O157 properties [26] to inhibit the attachment of *E. coli* O157:H7 to HEp-2 cells and to bovine colon which is the primary site of colonization. Adherence assays by using cell lines may constitute a simple, interesting, and well-dimensioned model for conducting studies about the behavior of colicinogenic *E. coli* in the inhibition of the pathogenic strain colonization. There are studies that investigated the adherence of *E. coli* O157:H7 to cells and to organ explants *ex vivo*. In concordance with our findings, Dibb-Fuller et al. [30] showed that *E. coli* O157:H7 adhered to HEp-2 cells and with the highest number when compared with *E. coli* K12. In a study that tested the ability of lactobacilli to antagonize the biological effects of EHEC, Hugo et al. [31] could demonstrated, that a preincubation of monolayers with lactobacilli before inoculation with a clinical isolate of EHEC prevented detachment of eukaryotic cells and minimizes both F-actin rearrangement and morphological alterations. In our study, we found that when HEp-2 cells were inoculated with colicinogenic *E. coli *and* E. coli *O157:H7, the adherence of these serotypes showed a marked reduction in comparison to when only pathogenic strain was inoculated. Girard et al. [32] demonstrated that the infection of an IVOC model from terminal ileon, terminal colon, and terminal rectum with *E. coli* O157:H7 resulted in a consistent pattern of colonization, characterized by scattered foci of intimately adherent bacteria in the terminal ileum and colon, whereas foci covering a larger surface of the epithelium were frequently observed in the terminal rectum and *E. coli* O157 were also found deep in the crypts in the terminal rectum. Phillips et al. [20] showed that O157:H7 caused attaching/effacing lesions on bovine mucosa, while Baehler and Moxley [19] inoculated bovine colon and rectum explants with *E. coli* O157:H7 and demonstrated that epithelial cells of inoculated explants developed A/E lesions at the bacterial attachment sites, providing evidence that the large intestinal mucosal epithelium may be a site of infection that contributes to carriage of *E. coli* O157:H7 in adult cattle. These results are consistent with ours, where we have shown that when the explants were inoculated with *E. coli* O157:H7, the strain was found attached to the epithelium of the colonic mucosa of the bovine species. Taking into account that colicinogenic *E. coli* was able to reduce the adherence of *E. coli* O157:H7 when both strains were inoculated on cell cultures and on bovine colonic explants, the impact of our study is that the colicinogenic strains could be used as a strategy to reduce cattle colonization with the pathogenic strain.

These are promising findings that allow thinking in the use of the colicinogenic *E. coli* as a potential probiotic strain and in the control of the colonization of bovine gastrointestinal tract with *E. coli* O157:H7. The colicinogenic strains may be used in the live animal on the farm before slaughter, thus reducing the entry of this pathogenic bacterium in the food chain. By this way, it may have the largest impact on improving beef safety and on the reduction of the possibilities of human infection.

## Figures and Tables

**Table 1:** Percentages of bacteria adhered to HEp-2 cells.

Strains	Inoculum (cfu mL^−1^)	% of adhered and recovered bacteria
* E. coli* O157:H7	10^5^	38.5
Colicinogenic *E. coli *	10^5^	61.5
* E. coli* O157:H7	10^5^	3.6
Colicinogenic *E. coli *	10^6^	96.4
* E. coli* O157:H7	10^5^	100.0
Colicinogenic *E. coli *	10^5^	100.0
